# Burnout among oncology nurses and technicians in Morocco: Prevalence, risk factors, and structural equation modeling

**DOI:** 10.18632/oncoscience.623

**Published:** 2025-07-31

**Authors:** Imane Errami, Saber Boutayeb, Hassan Errihani

**Affiliations:** ^1^Faculty of Medicine and Pharmacy, University Mohammed V of Rabat, Rabat 10100, Morocco

**Keywords:** burnout, oncology, healthcare professionals, risk factors

## Abstract

Background: Burnout is an increasing concern in oncology, with significant implications for staff well-being and the quality of care delivery. This study aimed to determine the prevalence of burnout among oncology healthcare professionals in Morocco, primarily nurses and technicians, and to identify associated sociodemographic and occupational factors.

Materials and Methods: A cross-sectional study was conducted from September to December 2024 at the National Institute of Oncology in Rabat. Ninety-one healthcare professionals completed the Maslach Burnout Inventory–Human Services Survey (MBI-HSS). Structural Equation Modeling (SEM) was used to explore the interrelationships among the three burnout dimensions.

Results: Severe burnout was identified in 61.5% of participants. Emotional exhaustion was the most affected dimension (70.4% moderate or high), followed by depersonalization (57.1%). While 50.5% reported high levels of personal accomplishment, 24.2% reported low fulfillment. Higher levels of burnout were associated with younger age, female gender, nursing roles, and night shift work. SEM analysis confirmed the central role of emotional exhaustion in predicting both increased depersonalization (β = 0.524, *p* = 0.002) and reduced personal accomplishment (β = –0.820, *p* = 0.003).

Discussion and Conclusion: Burnout is highly prevalent among oncology healthcare professionals in Morocco, particularly among young female nurses. Key contributing factors include age, gender, level of seniority, and shift schedules. Participants emphasized the importance of reducing workload, improving working conditions, adjusting salaries, and promoting continuing education. Institutional interventions are urgently needed to address burnout and protect healthcare worker well-being.

## INTRODUCTION

The World Health Organization (WHO) defines burnout as a work-related syndrome resulting from chronic workplace stress that has not been effectively managed. It is increasingly recognized as a public health issue, particularly in healthcare settings, where it affects both the well-being of providers and the quality of patient care [1].

According to Maslach’s model, burnout comprises three interconnected dimensions: emotional exhaustion, depersonalization, and a reduced sense of personal accomplishment. Together, these factors can negatively impact healthcare professionals’ mental health, diminish care quality, impair hospital performance, and compromise patient safety [2].

Global studies have reported alarming burnout rates among medical and paramedical personnel. Recent estimates suggest that more than 50% of physicians and nearly 60% of hospital nurses experience burnout [3]. These figures are particularly high in emotionally demanding specialties such as oncology, where burnout rates have reached 71% among young oncologists in Europe and 45% in the United States [4].

In the Middle East and North Africa (MENA) region, the multicenter BOMENA study highlighted similarly high levels of burnout among oncology professionals [5]. Factors contributing to this include excessive workloads, limited organizational support, constant exposure to patient suffering and death, and the psychological toll of managing complex, life-threatening conditions.

Several studies in Morocco have echoed these findings, documenting elevated stress levels among healthcare professionals, particularly those working in anesthesia, intensive care, and COVID-19 units [3, 4]. These results point to the importance of working conditions and structural limitations as major contributors to burnout.

Despite their critical roles in patient care, oncology nurses and health technicians remain underrepresented in the burnout literature. This is particularly concerning given the frequent staff shortages and sustained workloads that characterize oncology settings.

Recent evidence suggests that oncology professionals may experience psychological stress levels equal to or exceeding those of frontline clinicians. This may stem from their continuous exposure to patient suffering, the cumulative emotional burden of care delivery, and their expanded administrative responsibilities [6].

Given the limited research focusing on this population, there is a pressing need to understand the specific mechanisms underlying burnout in oncology support staff. Such insight is essential for developing targeted interventions that protect staff well-being, maintain high standards of care, and support better patient outcomes.

This study aimed to estimate the prevalence of burnout among healthcare professionals at the National Institute of Oncology (INO) in Rabat, Morocco—a leading national center for cancer care. Additionally, we sought to identify key sociodemographic and occupational risk factors. To achieve these objectives, we used the Maslach Burnout Inventory (MBI) and applied Structural Equation Modeling (SEM), a rarely used but powerful method for analyzing the complex relationships among burnout dimensions and their predictors. This approach allows for a deeper understanding of the pathways contributing to burnout and supports the development of tailored, evidence-based prevention strategies.

## RESULTS

### Sociodemographic and professional characteristics

Of the 200 questionnaires distributed, 91 were returned (response rate: 45.5%). The mean age of participants was 34 ± 5 years, with a female predominance (64.8%). More than half of the respondents were married (54.9%), and 43.8% reported no dependent children.

Professionally, 63.7% worked as nurses, 18.5% as health technicians, and 17.6% held administrative positions. Experience in oncology varied: 34.1% had worked for more than five years, and 28.6% for more than ten years. Regarding work organization, 72.5% of professionals worked mainly during the daytime, while 34.1% performed between five and ten night shifts per month (Table 1).

**Table 1:** Sociodemographic and professional characteristics of participants (*N* = 91)

Variables	*N* (%)
**Gender**	
Female	59 (64.8)
Male	32 (35.2)
**Age range**	
20 to 35 years	49 (53.8)
35 to 45 years	36 (39.6)
Over 45 years	6 (6.6)
**Professional profile**	
Nurse	58 (63.7)
Health technician	33 (36.3)
**Department**	
Medical inpatient department	30 (33.0)
Day hospital	16 (17.6)
Radiotherapy department	11 (12.1)
Administrative department	8 (8.8)
Consultation department	7 (7.7)
Operating room	7 (7.7)
Surgical department	6 (6.6)
Emergency department	2 (2.2)
Radiology department	2 (2.2)
Intensive care unit	2 (2.2)
**Experience in oncology**	
Between 5 and 10 years	31 (34.1)
More than 10 years	26 (28.6)
Between 1 and 5 years	19 (20.9)
Less than a year	15 (16.5)
**Work organization**	
Days more than nights	66 (72.5)
Nights more than days	13 (14.3)
Equal days and nights	12 (13.2)
**Number of on-call duties per month**	
None	40 (44.0)
Between 5 to 10 duties	31 (34.1)
Less than 5 duties	13 (14.3)
More than 10 duties	7 (7.7)

### Health conditions and lifestyle

Data analysis reveals that 24.2% of participants suffered from a chronic disease. Sleep was generally insufficient: 30.8% slept less than five hours per night and 37.4% between five and six hours. A majority of participants (71.4%) did not engage in regular physical activity.

Health behaviors were generally favorable: 82.4% did not smoke, 91.2% did not consume alcohol, and none reported drug use. In terms of mental health, 24.2% of respondents reported a history of depression, and 19.8% had consulted a mental health professional (Table 2).

**Table 2:** Health characteristics and lifestyle of participants (*N* = 91)

Variables	*N* (%)
**Chronic diseases**	
No	69 (75.8)
Yes	22 (24.2)
**Hours of sleep per night**	
5−6 hours	34 (37.4)
4−5 hours	28 (30.8)
More than 6 hours	21 (23.1)
Less than 4 hours	8 (8.8)
**Sport practice**	
No	65 (71.4)
Yes	26 (28.6)
**Family/friend outings**	
1 to 5 times	61 (67.0)
None	29 (31.9)
More than 5 times	1 (1.1)
**Coffee consumption (cups/day)**	
0	36 (39.6)
1	41 (45.1)
2	10 (11.0)
3 and more	4 (4.4)
**Alcohol consumption**	
0	83 (91.2)
1 glass/month	8 (8.8)
**Tobacco consumption**	
0	75 (82.4)
Less than 10 cigarettes/day	16 (17.6)
**Self-medication**	
None	64 (70.3)
Antidepressants	13 (14.3)
Anxiolytics	9 (9.9)
Hypnotics	3 (3.3)
Others	2 (2.2)
**Psychiatric history**	
No	59 (64.8)
Depression	22 (24.2)
Anxiety disorder	8 (8.8)
Others	2 (2.2)
**Consultation with psychiatrist/psychologist**	
No	73 (80.2)
Yes	18 (19.8)
**Suicidal thoughts**	
No	80 (88.9)
Occasionally	8 (8.9)
Frequently	2 (2.2)

### Work environment and occupational stress

Regarding the workplace environment, 15.4% of participants reported experiencing verbal abuse, and 34.1% had encountered professional conflict. Only 11% expressed complete satisfaction with their work climate, while 54.9% were dissatisfied with their salary.

The most frequently reported sources of stress included work overload (49.5%), lack of resources (44%), and administrative pressure (38.5%). Common coping strategies were recreational outings or travel (16.5%) and physical activity (11%). Notably, 9.9% reported using no stress management strategy at all (Table 3).

**Table 3:** Characteristics of work environment and stress factors (*N* = 91)

Variables	*N* (%)
**Victim of verbal violence**	
No	77 (84.6)
Yes	14 (15.4)
**Victim of physical violence**	
No	90 (98.9)
Yes	1 (1.1)
**Workplace conflicts (last 3 weeks)**	
No	60 (65.9)
Yes	31 (34.1)
**Work climate satisfaction**	
Satisfied	10 (11.0)
Moderately satisfied	67 (73.6)
Not satisfied	14 (15.4)
**Financial satisfaction**	
Satisfied	2 (2.2)
Moderately satisfied	39 (42.9)
Not satisfied	50 (54.9)
**Autonomous work management**	
Yes	49 (53.8)
No	42 (46.2)
**Interpersonal relationships**	
Satisfactory	34 (37.4)
Moderately satisfactory	49 (53.8)
Unsatisfactory	7 (7.7)
**Recognition at work**	
No	30 (33.0)
Patients	16 (17.6)
Supervisors and Colleagues	6 (6.6)
Others	39 (42.8)
**Main stress factors**	
Work overload	45 (49.5)
Lack of resources	40 (44.0)
Administrative pressure	35 (38.5)
Devaluation of profession	30 (33.0)

### Awareness of burnout and prevention strategies

While 78% of participants were familiar with the concept of burnout, few institutional preventive measures were reportedly in place. A high level of dissatisfaction was evident, with 80.2% indicating a desire to change professions.

Participants recommended several strategies for preventing burnout: workload reduction (49.5%), improvement of working conditions (44%), financial incentives (38.5%), continuing education (33%), and professional recognition (27.5%) (Table 4).

**Table 4:** Burnout perception and prevention: Knowledge, career change intent, and identified prevention factors (*N* = 91)

Variables	*N* (%)
**Knowledge of burnout**	
Yes	71 (78.0)
No	20 (22.0)
**Career change intent**	
No	18 (19.8)
Yes	73 (80.2)
**Perceived factors to prevent burnout**	
Reduction in workload	45 (49.5)
Improvement of working conditions	40 (44.0)
Financial motivation	35 (38.5)
Continuing education	30 (33.0)
Recognition	25 (27.5)
Presence of psychologists	20 (22.0)
Others (clubs, stress management, travel, etc.)	15 (16.5)

### Burnout prevalence and levels

According to the Maslach Burnout Inventory, 61.5% of participants experienced severe burnout, 27.5% had moderate burnout, and only 11% reported low levels.

**Figure 1:**
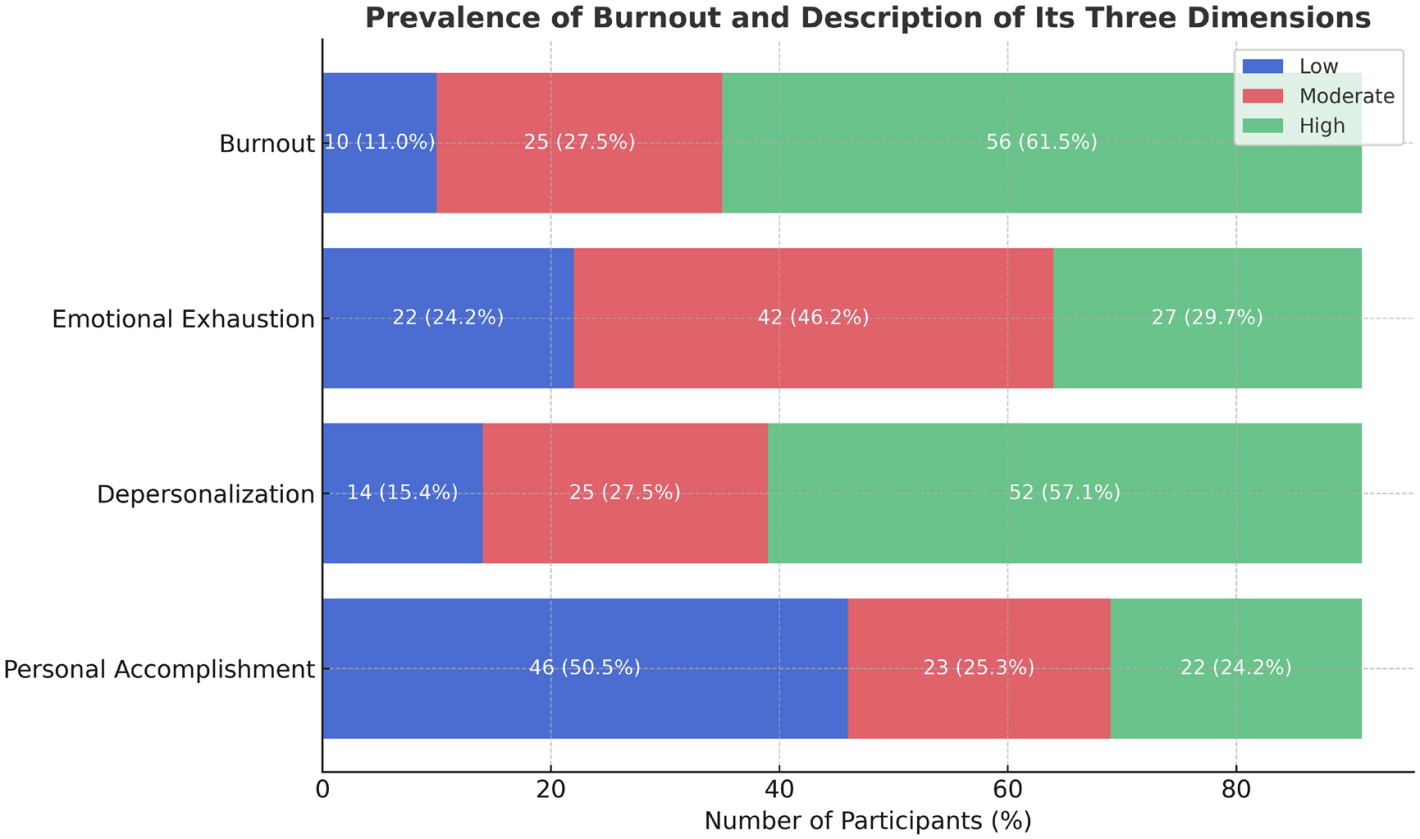
Distribution of burnout levels according to the three MBI dimensions (*N* = 91).

Breakdown by dimension:

*Emotional Exhaustion (EE)*: 46.2% had moderate levels and 24.2% had high levels (total of 70.4% moderate to high).
*Depersonalization (DP)*: 57.1% scored high, indicating emotional detachment in patient care.
*Personal Accomplishment (PA)*: While 50.5% reported high accomplishment, 24.2% reported low professional fulfillment (Figure 1).

### Factors associated with burnout dimensions

Bivariate analyses revealed several statistically significant associations:

*High Depersonalization* was more frequent among younger professionals (20–35 years) (*p* = 0.024), nurses (*p* = 0.021), those working alternating shifts (*p* = 0.040), and those who consumed alcohol (*p* = 0.001) or tobacco (*p* = 0.001).
*High Emotional Exhaustion* was more common among nurses (*p* = 0.030), professionals with 1–5 years of experience (*p* = 0.002), alcohol users (*p* = 0.015), tobacco users (*p* = 0.009), and those who had consulted a mental health professional (*p* = 0.035).
*Low Personal Accomplishment* was significantly associated with female gender (*p* = 0.001), younger age (*p* = 0.011), frequent night shifts (*p* = 0.021), and alternating shift schedules (*p* = 0.016) (Table 5).

**Table 5:** Sociodemographic and professional factors significantly associated with burnout dimensions (*N* = 91)

Dimension	Associated factors (*p*-value; Cramer’s V)
High Depersonalization	Young age (20–35 years): (*p* = 0.024; V = 0.22) Nursing profession (vs. technician): (*p* = 0.021; V = 0.18) Work in alternating shifts (day/night posts): (*p* = 0.040; V = 0.23) Alcohol consumption: (*p* = 0.001; V = 0.31) Smoking :(*p* = 0.001; V = 0.30)
High Emotional Exhaustion	Nursing profession: (*p* = 0.030; V = 0.22) Experience 1–5 years (vs. >5 years): (*p* = 0.002; V = 0.34) Alcohol consumption: (*p* = 0.015; V = 0.27) Smoking :(*p* = 0.009; V = 0.29) Psychiatric consultation: (*p* = 0.035; V = 0.26)
**Low Personal Accomplishment**	**Female gender: (*p* = 0.001; V = 0.338)** **Experience 1–5 years: (*p* = 0.011; V = 0.27)** **≥5 night shifts per month: (*p* = 0.021; V = 0.25)** **Alternating work schedules: (*p* = 0.016; V = 0.25)**

*p*-values represent the significance level of the association. Cramer’s V indicates the strength of association.

### Structural equation modeling (SEM) of burnout dimensions

Structural Equation Modeling revealed the following relationships among the three burnout dimensions:

- *Emotional Exhaustion* was a significant predictor of *Depersonalization* (β = 0.524; *p* = 0.002).
- *Emotional Exhaustion* also predicted *Reduced Personal Accomplishment* (β = –0.820; *p* = 0.003).
- The direct relationship between *Depersonalization* and *Personal Accomplishment* was not statistically significant (*p* = 0.096).

**Figure 2:**
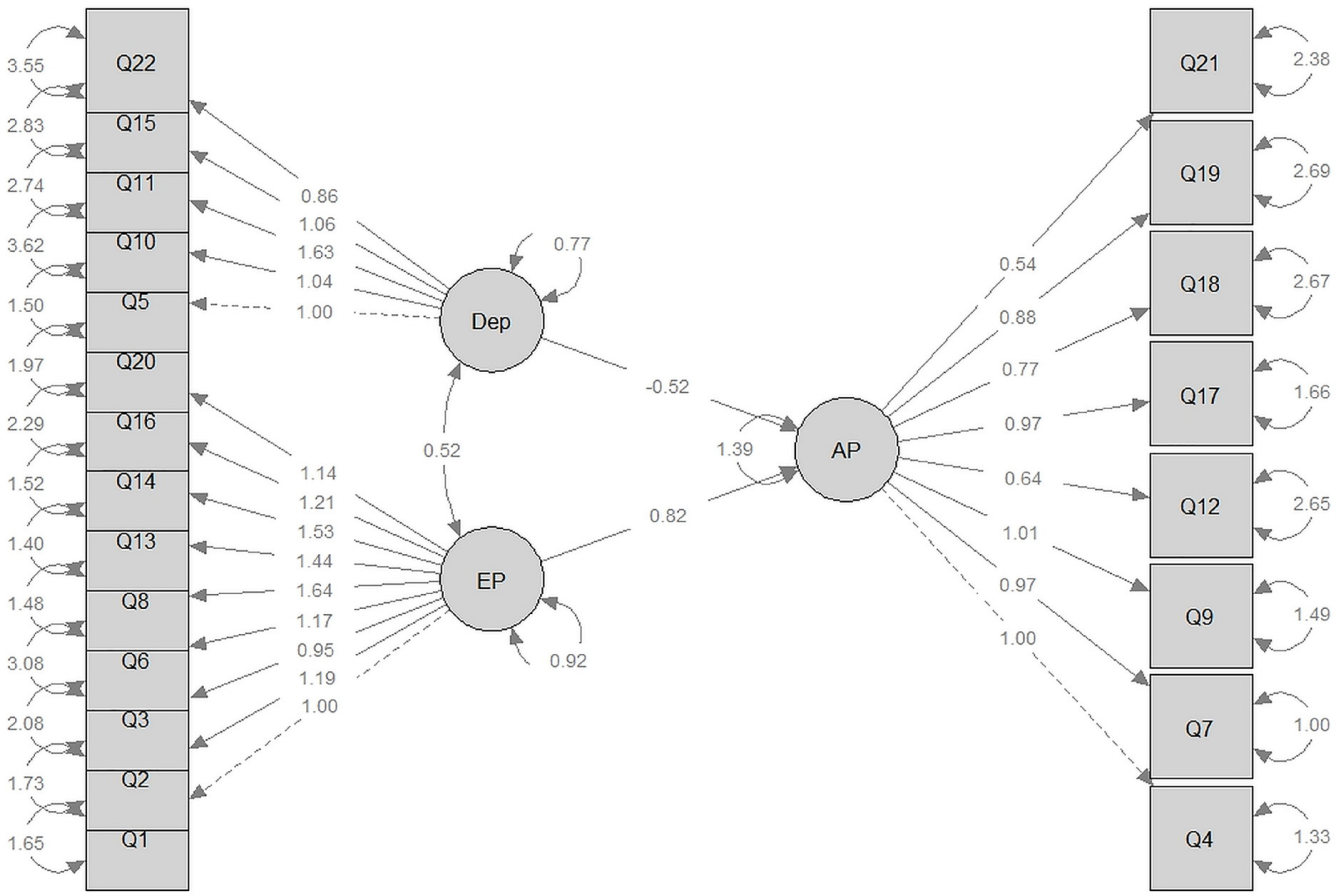
Modeling relationships between burnout dimensions using structural equation modeling (SEM). Abbreviations: EE: Emotional Exhaustion; DP: Depersonalization; PA: Personal Accomplishment.

While SEM provided valuable insights, model fit indices (CFI = 0.798; RMSEA = 0.088) did not meet conventional cutoffs for excellent fit (e.g., CFI ≥0.90). This may be due to limited sample size or omitted variables. Future research should consider model refinements or alternative approaches, such as path analysis (Figure 2).

## DISCUSSION

### Profile of healthcare professionals and vulnerability to burnout

The majority of respondents in this study were nurses and health technicians involved in direct patient care, consistent with prior findings identifying frontline oncology staff as particularly vulnerable to burnout due to high clinical demands and limited recovery time [7]. This is especially relevant in oncology, where caregiving involves sustained emotional engagement with patients and their families.

Our results align with previous studies showing that female-dominated professions, such as nursing, are at increased risk of emotional exhaustion. This is likely due to repeated interpersonal stressors, long hours, and limited organizational support [8]. Younger professionals and those with fewer years of experience were also more likely to report higher burnout levels. This is consistent with findings by Stimpfel et al., who reported that younger nurses working long shifts were more susceptible to burnout and early career dissatisfaction [9].

### Work environment and occupational stress

Consistent with the literature, our findings highlight shift work, workload pressure, and administrative demands as key contributors to burnout [10, 11]. Many participants expressed dissatisfaction with their remuneration and workplace climate, and over 80% indicated an intention to leave their profession—an alarming figure that mirrors international findings linking burnout to high turnover intentions [11].

Extended work hours, limited time off, and organizational inefficiencies have been shown to erode resilience and increase emotional fatigue [12, 13]. These structural issues contribute to burnout by creating an imbalance between job demands and available resources, as outlined in Maslach and Leiter’s “Areas of Worklife” model [2].

### Analysis of burnout dimensions

*Emotional Exhaustion*, reported by 70.4% of participants at moderate to high levels, emerged as the most prevalent dimension. This aligns with prior research emphasizing its central role in burnout, particularly in emotionally demanding environments such as oncology [14, 15]. Elbarazi et al. similarly found that emotional exhaustion is highly prevalent among healthcare professionals in Arab countries, often exceeding rates reported in Western contexts [16].

*Depersonalization* was elevated in 57.1% of respondents. This finding reflects emotional detachment and reduced empathy in caregiver-patient relationships, and supports prior observations by Shanafelt et al., who identified depersonalization as a core feature of burnout among physicians [17]. Interestingly, in our sample, depersonalization was associated with alternating shifts and younger age, though not with gender—consistent with meta-analyses indicating minimal gender differences for this dimension [8].

*Personal Accomplishment* was preserved in approximately half the participants, but 24.2% reported low fulfillment. Lower accomplishment scores were significantly associated with female gender, early career status, and shift work. This trend supports earlier findings that job satisfaction and career longevity are negatively impacted by inadequate recognition and overwhelming workloads [12].

### Structural modeling and pathways of burnout

In our study, gender was identified as a significant factor influencing burnout, with women exhibiting lower levels of personal accomplishment compared to men. This finding is consistent with previous research. Abusanad et al. reported that female oncologists had a higher prevalence of emotional exhaustion and lower personal accomplishment, suggesting gender-related vulnerabilities in occupational well-being. Similarly, Purvanova and Muros demonstrated through meta-analysis that women are slightly more emotionally exhausted than men, while men show higher levels of depersonalization, further supporting the gendered nature of burnout symptoms [5–19].

Age and seniority were also shown to significantly influence burnout in our study, with younger and less experienced professionals experiencing higher emotional exhaustion and depersonalization. These findings are in line with those of Abusanad et al., who observed that younger oncology professionals were particularly susceptible to burnout, potentially due to evolving professional skills and lack of mentoring support. In addition, Stimpfel et al. highlighted that extended shift lengths and the resulting workload pressure disproportionately affected younger nurses, contributing to elevated burnout levels and increased intentions to leave the profession [5–9].

The analysis revealed that alternating shift work is closely associated with heightened depersonalization and diminished personal accomplishment among healthcare professionals. This pattern is supported by the findings of Wisetborisut et al., who observed that shift workers reported significantly elevated levels of emotional exhaustion and burnout compared to non-shift workers. Although their study showed a stronger association with emotional exhaustion, the link between shift work and challenges in maintaining personal accomplishment was suggested [10].

Similarly, Benard Gisilanbe emphasized that irregular and extended shift patterns increase the risk of stress and fatigue among nurses, leading to manifestations of depersonalization, emotional exhaustion, and reduced personal accomplishment. These findings collectively highlight that alternating shifts may considerably exacerbate psychological strain and professional dissatisfaction, underscoring the critical need for organizational interventions to mitigate burnout among healthcare providers [17].

In accordance with our results, which indicate that excessive workload reinforces emotional exhaustion, reduces the sense of personal accomplishment, and promotes a high intention of professional departure among 80% of respondents, the findings of Hlubocky et al. confirm these observations. They emphasize that excessive patient load, prolonged working hours, and increased administrative responsibilities are major contributors to oncologist burnout. Furthermore, burnout is linked to increased turnover, reduced clinical hours, absenteeism, and early retirement, creating a vicious cycle that exacerbates staff shortages and occupational stress [18].

### Burnout prevention strategies: Alignment with international recommendations

In our study, participants prioritized the reduction of workload (49.5%), the improvement of working conditions (44%), the revaluation of salaries (38.5%), and the promotion of continuing education (33%) as key measures to prevent burnout. These findings demonstrate a strong concordance with international recommendations, particularly regarding the emphasis on workload management and workplace environment enhancement [12].

However, a slight discordance was observed concerning salary revaluation and continuing education, which, although recognized, are less prominently prioritized compared to organizational and systemic interventions in broader international guidelines [12].

Organizational interventions such as workload revision, schedule adjustment, and promotion of flexibility in working conditions are identified as key strategies for preventing professional burnout. These priorities are emphasized in both our findings and in official recommendations from the French National Academy of Medicine [19].

Similarly, Shanafelt and Noseworthy, in their Strategy 7 titled Promote Flexibility and Work-Life Integration, explicitly recommend the implementation of flexible work schedules and the limitation of workload as essential organizational levers to enhance physician well-being and reduce burnout. These measures are identified among nine key organizational strategies aimed at fostering engagement and promoting a healthier work environment [20].

In light of our findings, targeted measures such as mentoring programs, workload and schedule stabilization strategies, and broader organizational changes appear necessary to address burnout-related disparities. This is consistent with the work of Panagioti et al., who emphasized that interventions enhancing teamwork, mentoring, and leadership skills are particularly effective among younger and high-risk physicians. Additionally, organization-directed changes focusing on workload and scheduling adjustments have been shown to achieve greater reductions in burnout than physician-directed interventions [21].

Improving working conditions remains essential to mitigating burnout, particularly by fostering a positive team climate, ensuring managerial support, and enhancing access to material and professional resources. Maslach and Leiter’s “Areas of Worklife” model underscores that burnout is primarily promoted by mismatches between six critical domains, namely workload, control, reward, community, fairness, and values, and the individual expectations. A greater alignment between these domains and personal expectations is associated with higher levels of engagement, whereas substantial misalignments considerably increase the risk of burnout [2].

The need for both financial and moral recognition expressed by healthcare professionals in our study aligns partially with existing literature. Maslach and Jackson highlighted the protective effect of moral recognition, including positive feedback and the reinforcement of personal accomplishment, against burnout. Although their study did not specifically address financial recognition, their findings underscore the critical role of acknowledgment and reward mechanisms in promoting professional engagement [13].

Although not explicitly prioritized in interventions reviewed by West et al., strategies focusing on mindfulness have demonstrated effectiveness in reducing emotional exhaustion and overall burnout among physicians. Their systematic review also emphasized the critical importance of maintaining a healthy work-life balance and ensuring psychological support to mitigate burnout. However, the specific role of continuing education in reinforcing professional mastery was not addressed in their findings [16].

Participants’ responses in our study demonstrate an intuitive understanding of the multilevel nature of burnout interventions, encompassing individual, organizational, and institutional domains. This perspective aligns closely with contemporary models and is supported by the findings of Panagioti et al., who classified interventions into physician-directed strategies, such as mindfulness training, communication skills, and coping mechanisms, and organization-directed strategies, including schedule adjustments, workload reduction, improved teamwork, and participatory decision-making processes [22].

Furthermore, their conclusion reinforces the need for systemic approaches, stating that “burnout is a problem of the whole healthcare organization rather than of individuals,” thus emphasizing the necessity of coordinated interventions across multiple levels to effectively address burnout among healthcare professionals [21].

The formal recognition of burnout as an occupational phenomenon in the International Classification of Diseases, 11th Revision (ICD-11) by the World Health Organization in 2019 further reinforces the need for comprehensive, system-wide strategies [22].

### Implications and future directions

This study highlights the urgent need for institutional policies to address burnout among oncology staff in Morocco. High burnout levels not only compromise individual well-being but may also lead to reduced clinical performance, lower patient satisfaction, and increased staff attrition. Interventions must target both systemic organizational reforms and support for individual coping strategies.

Additionally, the self-selection sampling method and response rate of 45.5% may introduce bias. The findings may not generalize to all oncology professionals.

Future research should incorporate longitudinal designs and multivariate analyses to better understand the causal pathways of burnout and evaluate the impact of preventive measures. There is also a need for broader sampling beyond a single institution to enhance generalizability.

## MATERIALS AND METHODS

### Study design and setting

A cross-sectional observational study with both descriptive and analytical aims was conducted at the National Institute of Oncology (INO) “Sidi Mohamed Ben Abdellah” in Rabat, Morocco. This public reference institution, affiliated with the Ibn Sina University Hospital Center, specializes in multidisciplinary cancer management. Data collection occurred between September and December 2024 and encompassed all clinical units, including medical oncology, surgical oncology, radiotherapy, brachytherapy, chemotherapy, palliative care, and intensive care departments.

### Population and sampling

The study targeted mid-level oncology healthcare staff, primarily nurses and technicians, involved in direct patient care. Inclusion criteria required a tenure of at least six months at the INO and informed consent to participate. Staff on extended leave or absent during the data collection period were excluded.

A non-probabilistic, self-selection sampling method was employed. Out of 200 distributed invitations, 91 usable questionnaires were collected, yielding a response rate of 45.5%. The final sample consisted of 64.8% women and 35.2% men, aged 24 to 45 years (mean: 34 ± 5 years). Nurses comprised 63.7% of respondents, followed by health technicians (18.5%) and administrative staff (17.6%). More than half (53.8%) had between 1 and 5 years of experience, and 46.2% worked alternating day/night shifts.

### Tools and data collection

Data were collected via a structured, self-administered online questionnaire (Google Forms), distributed through email and internal messaging platforms (WhatsApp). Participation was voluntary, anonymous, and confidential.

The questionnaire included three sections:

*Sociodemographic and professional characteristics*: Gender, age, family status, seniority, department, role, work schedule and duration, number of on-call duties.
*Health and lifestyle*: Chronic illnesses, sleep quality, physical activity, substance use (tobacco, alcohol, coffee, psychotropic drugs), and use of psychological or psychiatric support.
*Stress and burnout status*: Job satisfaction, perceived recognition, occupational stress. Burnout was assessed using the Maslach Burnout Inventory – Human Services Survey (MBI-HSS), which measures three dimensions:
  *Emotional Exhaustion* (EE, 9 items, max score 54)
  *Depersonalization* (DP, 5 items, max score 30)
  *Personal Accomplishment* (PA, 8 items, max score 48).

Burnout severity was categorized based on MBI subscale thresholds, using the standard approach:

- *High burnout*: EE ≥27 and DP ≥10 and PA ≤33
- *Moderate burnout*: At least one dimension in the moderate range (EE: 17–26, DP: 6–9, PA: 34–39) without meeting criteria for high burnout
- *Low burnout*: EE ≤16 *and* DP ≤5 *and* PA ≥40.

### Statistical analysis

Analyses were performed using SPSS software (version 21). Qualitative variables were summarized as frequencies and percentages, while quantitative variables were described using means ± standard deviations.

Bivariate analyses explored associations between participant characteristics and burnout levels (dichotomized as “high” vs. “low/moderate”) using χ² tests or Fisher’s exact test, and Student’s *t*-tests. Statistical significance was set at *p* ≤ 0.05. The strength of categorical associations was assessed using Cramer’s V with 95% confidence intervals.

Structural Equation Modeling (SEM) was conducted with the lavaan package in R to examine the structural relationships among burnout dimensions. SEM was selected due to its capacity to simultaneously analyze complex relationships between latent and observed variables while accounting for measurement errors. The hypothesized model posited emotional exhaustion as a predictor of depersonalization and personal accomplishment, with depersonalization potentially mediating personal accomplishment. Model fit was evaluated using χ², Comparative Fit Index (CFI), Tucker-Lewis Index (TLI), Root Mean Square Error of Approximation (RMSEA), and Standardized Root Mean Square Residual (SRMR).

## Abbreviations

COVID-19: coronavirus disease 2019
CFI: Comparative Fit Index
DP: Depersonalization
EE: Emotional Exhaustion
INO: National Institute of Oncology
MBI: Maslach Burnout Inventor
MENA: Middle East and North Africa
PA: Personal Accomplishment
RMSEA: Root Mean Square Error of Approximation
SRMR: Standardized Root Mean Square Residual
SEM: Structural Equation Modeling
TLI: Tucker-Lewis Index
WHO: World Health Organization
